# Effects of detraining on neuromuscular function and structural adaptations following once- or twice-weekly eccentric resistance training in older adults

**DOI:** 10.1007/s40520-024-02828-1

**Published:** 2024-08-22

**Authors:** Brett A. Baxter, Anthony W. Baross, Declan J. Ryan, Anthony D. Kay

**Affiliations:** Centre for Physical Activity and Life Sciences, Faculty of Art, Science and Technology, University of Northampton, Northamptonshire, NN1 5PH UK

**Keywords:** Training cessation, Neuromuscular regression, Lengthening contractions

## Abstract

**Background:**

Eccentric resistance training elicits greater preservation of training-induced muscular adaptations compared with other training modalities, however the detraining profiles of different training dosages remain unknown.

**Aims:**

To examine the detraining effects following once- or twice-weekly eccentric-specific resistance training in older adults.

**Methods:**

Twenty-one older adults (age = 70.5 ± 6.0 year) completed a 12-week detraining period following the 12-week eccentric training programmes with neuromuscular function and muscle structure assessed six (mid-detraining) and 12 (post-detraining) weeks following training cessation.

**Results:**

From post-training to post-detraining, no significant regression of the training-induced improvements (collapsed group data reported) occurred in power (0%), strength (eccentric = 0%, isometric = 39%), or explosive strength over numerous epochs (0–32%), resulting in values that remained significantly greater than at pre-training. However, significant regression in the improvements in muscle thickness (91%) and fascicle angle (100%) occurred, resulting in values that were not significantly greater than pre-training.

**Discussion:**

The limited regression in neuromuscular function following a 12-week detraining period has important implications for supporting eccentric exercise prescription in older adults who often face periods of inactivity. However, further work is required to develop an effective maintenance dosage strategy that preserves improvements in muscle structure.

**Conclusions:**

Eccentric resistance training elicits improvements in the neuromuscular function of older adults, which are sustained for at least 12 weeks after eccentric training cessation.

## Key Points

• Improvements in neuromuscular function were sustained for at least 12 weeks following training cessation, whereas significant regression in muscle structure occurred.

• Training frequency did not influence the regression profiles of training-induced adaptations.

• Further research is warranted to develop a maintenance dosage that preserves training-induced adaptations in muscle structure.

## Introduction

Neuromuscular decline as a process of aging is associated with compromised functional ability [1–3], the increased risk of falling [4], and escalating healthcare costs [5, 6]. There is wide consensus that resistance training is a highly efficacious strategy to increase muscle strength and size in older adults [7–9], with a position statement published from the National Strength and Conditioning Association reinforcing this contention [10] and recommendations from the National Institute of Care Excellence to include strength training as a fall prevention strategy [11]. Furthermore, resistance training is non-pharmacological and cost-effective, hence it is considered a key therapeutic intervention to counteract neuromuscular deterioration associated with aging. However, as older adults often face periods of reduced physical activity due to the greater prevalence of illness, injury, or hospitalization [12, 13], which may contribute towards the low adherence rates to two weekly strengthening training sessions [14], hence the longevity of the training-induced adaptations following training cessation needs to be examined when determining the efficacy of an intervention. This reduced physical activity accelerates neuromuscular deterioration [15], with as little as five days of bed rest reported to decrease lower-limb lean muscle mass and knee extension power by 4% and 15%, respectively [16]. Consequently, determining the potential regression profiles following the cessation of resistance training programmes in older adults needs to be established to improve clinical exercise prescription guidelines.

The period following training cessation is often referred to as “detraining”, whereby the temporal profile of training-induced adaptations can be assessed in the absence of a training stimulus to monitor the speed and magnitude of regression. The effect of detraining is particularly important in older adults, where as little as four weeks of detraining is sufficient to significantly reduce muscle strength [17]. These circumstances have been highlighted during the outbreak of COVID-19, whereby vulnerable individuals (often older adults) were recommended to shield for periods upwards of 12 weeks, which is a sufficient timeframe for muscle strength and/or size to significantly decline [18, 19] and could partly explain the elevated incidence of falls and fractures immediately post-lockdown [20]. Nonetheless, despite the importance of examining the longevity of training-induced adaptations following training cessation, the effects of detraining are examined to a lesser extent following the initial training programme in a range of important health-related characteristics in older adults. Thus, identifying strategies that not only promote substantial improvements in neuromuscular function, but are also able to maintain these improvements during a detraining period is of great importance for effective clinical practice.

Eccentric-only and traditional (cyclical concentric-eccentric contractions) resistance training have demonstrated a superior ability to preserve muscular adaptations when compared to concentric-only training [21–23], suggesting that active lengthening of muscle is pivotal to eliciting sustainable adaptations, even in the untrained contralateral limb [24]. Recent work from our laboratory [25, 26] confirmed the efficacy of eccentric-only resistance training to induce long-lasting improvements in lower-limb muscle function and size of older adults, indicated by significantly greater values than at pre-training following eight weeks of detraining. Furthermore, we also confirmed that comparable training-induced improvements in muscle function and size were elicited following once- or twice-weekly training [27], suggesting that weekly training frequency has minimal influence of muscular adaptations induced by eccentric resistance training in older adults, which may also be evident during detraining. Therefore, as the efficacy of eccentric-only training to preserve muscle function during a detraining period has been confirmed but the influence of training frequency on detraining profiles remains unknown, the aim of this study was to examine the effects of a 12-week detraining period on neuromuscular characteristics in older adults who had previously partaken in a once- or twice-weekly 12-week eccentric-only resistance training programme. It was hypothesized that (i) no significant regression in metrics of neuromuscular function (power, maximum strength, and explosive strength) and structure (muscle thickness and fascicle angle) would occur during the detraining period, and (ii) weekly training frequency would have no impact on regression.

## Methods

### Participants

Twenty-three community-dwelling older adults who were i) ≥ 60 years of age, ii) able to independently ambulate without walking aids, iii) free from any illness and/or medication that affected the neuromuscular system or balance, and iv) completed either a once- (G1X *n* = 12 [male *n* = 7, female *n* = 5], age = 71.1 ± 6.6 year, height = 1.7 ± 0.1 m, mass = 73.3 ± 13.7 kg) or twice-weekly (G2X *n* = 11 [male *n* = 5, female *n* = 6], age = 70.6 ± 5.5 year, height = 1.7 ± 0.1 m, mass = 71.0 ± 10.6 kg) 12-week eccentric resistance training programme [27], volunteered to take part in the detraining study. All participants provided written informed consent prior to partaking in the study with all data collected at the University Health and Performance Laboratory between December 2018 and June 2021. The University Research Ethics Committee approved the study with all procedures conducted in accordance with the Declaration of Helsinki. Sample size calculations have been reported in greater detail elsewhere [27], whereby additional participants were recruited (minimum sample size per group *n* = 8, recruited sample size per group *n* = 14) to account for attrition within the detraining segment of the study using the following statistical parameters: α = 0.05, β = 0.20, *d* = 1.56.

### Protocol overview

**Fig. 1:**
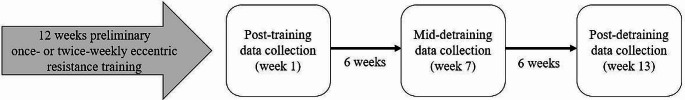
A schematic of the study design

The effects of a 12-week detraining period were examined following the cessation of the once- or twice-weekly 12-week eccentric resistance training programmes [27]. Data were collected in the present detraining study in week 1 (using post-training data collected from the previous training study), week six (mid-detraining), and week 12 (post-detraining) (Fig. 1). During the detraining period, participants were asked to return to their normal physical activity levels and were prohibited from taking part in new resistance training regimes. Habitual physical activity level data were collected using a commercially available accelerometer (Fitbit Inspire) but due to issues with compliance (discussed later), data could not be used for statistical analyses, therefore no daily physical activity level data were available within the present study. Data collection and analysis procedures have been outlined below but have been described in detail previously [27].

### Outcome measures

#### Vastus lateralis muscle structure

Participants were seated with a knee angle of 90° (180° = full extension) with B-mode ultrasonography used to assess in vivo muscle structure of the *vastus lateralis* (VL). The probe was positioned longitudinally and parallel to the direction of the muscle fascicles over the mid-point between the greater trochanter and lateral femoral condyle and manipulated until the deep and superficial VL aponeuroses were clearly visible. Subsequently, sonographs were exported and analysed using digitising software (ImageJ 1.46r, National Institutes of Health, Bethesda, MD, USA). Muscle thickness (mm) was defined as the distance between the superficial and deep aponeuroses whilst fascicle angle (°) was measured as the angle between the muscle fascicle and the deep aponeurosis. Three measures of VL muscle thickness and three VL fascicle angles were measured on two sonographs and the mean value of each was used for further analysis, which were secondary outcome measures.

#### Lower-limb power

A 10-repetition sit-to-stand (STS) test was used to assess lower-limb muscular power (W), which was a primary outcome measure. The test was performed twice with a 1-min rest between trials and the fastest trial used for subsequent analysis. Power was calculated as the quotient of work and time using the height of the chair (m), body mass (kg), and lower-limb length (distance from the greater trochanter to the lateral malleolus [m]) of each participant using previous methods [28]:

$$\:{\text{Power}}\:{\text{ = }}\:({\text{Body}}\:{\text{Mass}}\: \cdot \:{\text{g}} \cdot \:\:[{\text{Leg}}\:{\text{Length}}\:{\text{ - }}\:{\text{Chair}}\:{\text{Height}}]\: \cdot \:{\text{10}})\: \cdot \:{\text{Tim}}{{\text{e}}^{ - 1}}$$

where g = acceleration due to gravity (9.81 m·s^-2^), Time = time to complete 10 STS repetitions (s), 10 = ten repetitions.

#### Lower-limb contractile ability

Participants were then seated on the isokinetic dynamometer (Biodex System 3 Pro, IPRS, Suffolk, UK) with the hips flexed to 95° (180° = full extension) and the right knee flexed to 110°, i.e. where maximum knee extensor strength is produced in older adults [29, 30]. Participants performed three unilateral submaximal isometric contractions at 50 and 75% of perceived maximum followed by five explosive contractions as “*fast* and hard” as possible (with the emphasis on fast), with each contraction separated by 15 s rest. If a trial displayed signs of countermovement (checked visually) on the real-time torque-time trace, it was deemed invalid and the test was repeated.

Contractile impulse (the area under the curve of the torque-time trace [∫torque d*t*,]; N·m·s) and rate of torque development ([RTD] the slope of the torque-time trace [Δtorque‧Δtime^-1^]; N·m·s^-1^) were secondary outcome measures calculated from the onset of contraction over several epochs (0-100, 0-150, 0-200, 0-250, and 0-300 ms), alongside peak RTD (RTD_Peak_), defined as the steepest slope of the torque-time trace between 0 and 300 ms using a rolling 20-ms epoch [31]. The onset of muscular contraction (0 ms) was determined manually as this is more accurate than automated methods [32] using visual inspection of the inflexion point on the torque-time trace in a figure with a y-axis (torque) scale of ~ 1 N‧m and an x-axis (time) scale of ~ 200 ms [33]. The mean of the three most explosive trials (greatest RTD over all epochs) was used for subsequent analysis of RTD and contractile impulse [32].

Ramped maximal voluntary isometric contractions (MVIC) were performed over a 5-s epoch to measure maximal isometric knee extensor torque (N·m), which was a primary outcome measure. The MVICs were initiated from rest with participants instructed to reach maximum after ~ 3 s and continue to contract “as hard as possible” to enable a 2-s plateau and confirm that MVIC had been reached. Following a 1-min rest, another MVIC was performed until three valid trials were collected. The largest value of isometric torque from the three maximal trials was used for subsequent analysis. Torque data from these trials were directed to a personal computer (Elitebook, HP Inc., CA, USA) running AcqKnowledge software (v.4.4, Biopac) and then smoothed off-line in RStudio (v.1.0.153, RStudio, Inc., MA, USA) with a custom-written fourth-order, zero-lag Butterworth filter at 150 Hz [34].

Eccentric lower-limb force (N), which was a primary outcome measure was assessed on the same recumbent isokinetic stepper ergometer (Eccentron, Baltimore Therapeutic Equipment, Hanover, MD, USA) in which the training programmes were performed. Participants performed two submaximal warm-up sets of 12 repetitions (six per limb) at 25 and then 50% of their perceived maximal effort, followed by two sets of 12 maximal efforts, which were separated by a 1-min rest; the largest value of both limbs was used for subsequent analysis.

### Statistical analyses

Statistical analyses were performed using SPSS for Windows (v.28 IBM Corp., Armonk, NY, USA) with normality of distribution examined via Shapiro-Wilk tests and homogeneity of variance assessed via Levene’s or Mauchly’s tests. Data that did not meet the assumptions of normal distribution were transformed (square root or natural logarithm). Between-group differences of all outcome measures were assessed at post-training prior to any statistical analyses to determine whether covariates should be included within the analyses.

Data that satisfied the parametric assumptions were analysed using a two-way mixed-model ANOVA (time × 3 [post-training, mid-detraining, and post-detraining], group × 2 [G1X and G2X]) to examine within-, between-, and interaction-effects. Where a significant interaction effect was detected, simple main effects analyses (pairwise comparisons) were conducted using Bonferroni correction; if no interaction effect was revealed, the main effects of time and group were examined (using collapsed data). Subsequently, post-detraining values were compared to pre-training values via paired *t*-tests to examine overall effects of the training-detraining period on the training-induced improvements. The retainment of training-induced adaptations was calculated as the absolute percentage of the training-induced increase that was lost (100 - [post-detraining group mean - post-training group mean]/[post-training group mean – pre-training group mean] × 100), which have been reported for each training group. Within G1X, one participant did not attend mid-detraining data collection and one participant did not attend post-detraining data collection session due to illness and self-isolation from the COVID-19 virus, respectively, therefore statistical analyses were conducted on 21 participants (G1X *n* = 10, G2X *n* = 11). Standardized effect sizes were calculated to examine the magnitude of change with partial eta squared (η_p_^2^) and Cohen’s *d* (with 95% CI) calculated [35] for ANOVA and pairwise comparisons, respectively. Group data are reported as mean ± SE and change data are reported as mean ± SD. Statistical significance for all tests was accepted at *P* < 0.05.

## Results

At post-training, there were no significant between-group differences in any outcome measure. In addition, there were no significant interaction effects reported within the previous training study [27], demonstrating comparable adaptative profiles following the once- or twice-weekly training stimuli with increases reported in power (13%), isometric strength (17–36%), eccentric strength (40–50%), VL muscle thickness (9–18%), and VL fascicle angle (1.5-2.0°).

### Outcome measures

#### Vastus lateralis muscle structure

**Fig. 2:**
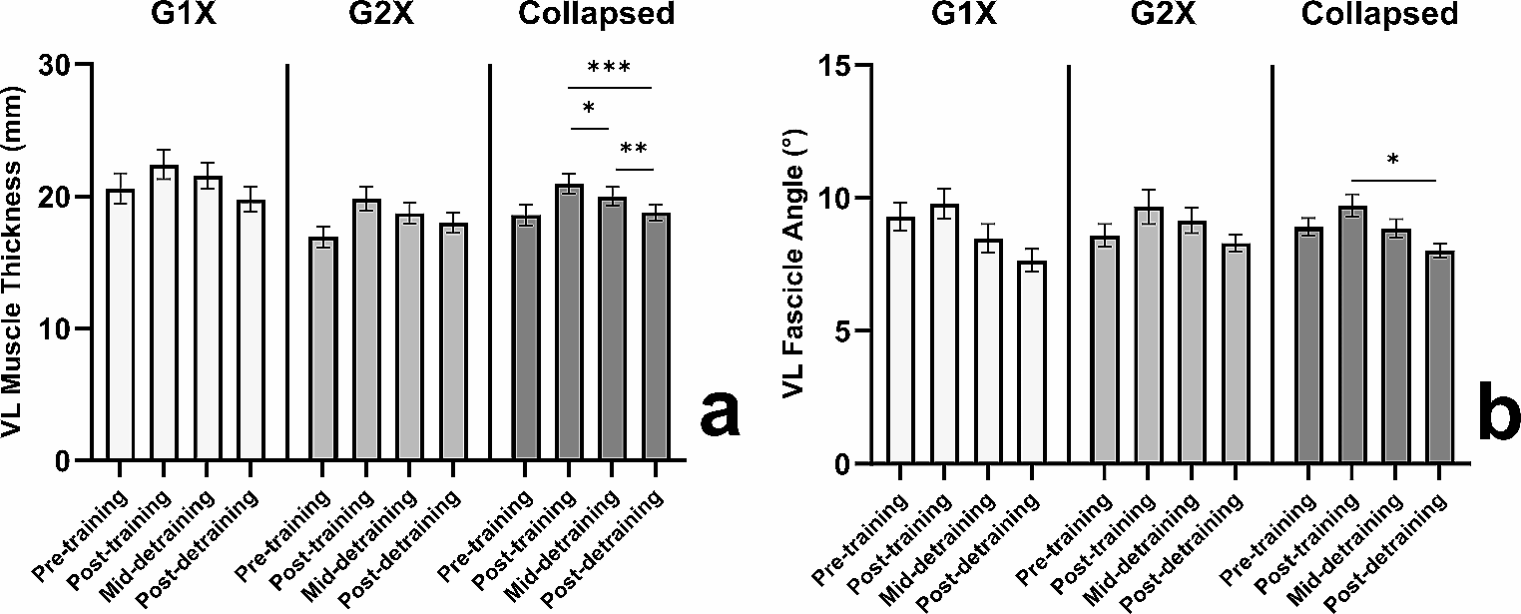
Mean ± SE for *vastus lateralis* (VL) muscle thickness (a) and fascicle angle (b) at pre-training, post-training, mid-detraining, and post-detraining in the once- (G1X) and twice-weekly (G2X) training groups, alongside data collapsed by training group; where * denotes a significant difference *P* < 0.05, ** denotes a significant difference *P* < 0.01, and *** denotes a significant difference *P* < 0.001

The two-way ANOVA revealed no significant interaction effect for muscle thickness (*F*_2, 36_ = 1.333, *P* = 0.276, η_p_^2^ = 0.069), with a significant main effect of time (*F*_2, 36_ = 24.859, *P* < 0.001, η_p_^2^ = 0.540) but not group (*F*_1, 18_ = 3.851, *P* = 0.065, η_p_^2^ = 0.176) detected. Data collapsed across groups revealed muscle thickness significantly decreased from post-training to mid-detraining (-4.3 ± 6.5% [-1.0 ± 1.5 mm]; *P* = 0.030, *d* = 0.67 [95% CI = 0.63, 0.71]), from mid-detraining to post-detraining (-5.7 ± 7.4% [-1.2 ± 1.5 mm]; *P* = 0.003, *d* = 0.81 [95% CI = 0.75, 0.86]), and post-training to post-detraining (-10.0 ± 7.5% [-2.2 ± 1.7 mm]; *P* < 0.001, *d* = 1.32 [95% CI = 1.23, 1.40]), indicative of substantial regression during the training period. In fact, 12 weeks of detraining lead to 91% of the training-induced increases in muscle thickness being lost, resulting in values not significantly different to pre-training (collapsed group data = 2.5 ± 12.5% [0.2 ± 2.4 mm]; *P* = 0.686, *d* = 0.09 [95% CI = 0.08, 0.10]), see Fig. 2a.

No significant interaction effect was revealed for fascicle angle (*F*_2, 36_ = 0.429, *P* = 0.655, η_p_^2^ = 0.023), with a significant main effect of time (*F*_2, 36_ = 6.489, *P* = 0.004, η_p_^2^ = 0.265) but not group (*F*_1, 18_ = 0.774, *P* = 0.391, η_p_^2^ = 0.041) detected. Data collapsed across groups revealed no significant reduction in fascicle angle from post-training to mid-detraining (-0.9 ± 2.4°; *P* = 0.368, *d* = 0.35 [95% CI = 0.33, 0.38]) or mid-detraining to post-detraining (-0.8 ± 1.5°; *P* = 0.115, *d* = 0.51 [95% CI = 0.48, 0.55]), however a significant reduction was detected from post-training to post-detraining (-1.7 ± 2.3°; *P* = 0.012, *d* = 0.73 [95% CI = 0.68, 0.78]). While a six-week epoch was not sufficient to reveal significant regression, twelve weeks of detraining lead to 100% of the training-induced increases in fascicle angle being lost, again indicative of substantial regression during the detraining period and resulting in values not significantly different to pre-training (collapsed group data = -0.9 ± 2.0°; *P* = 0.057, *d* = 0.45 [95% CI = 0.42, 0.48]), see Fig. 2b.

#### Lower-limb power

**Fig. 3:**
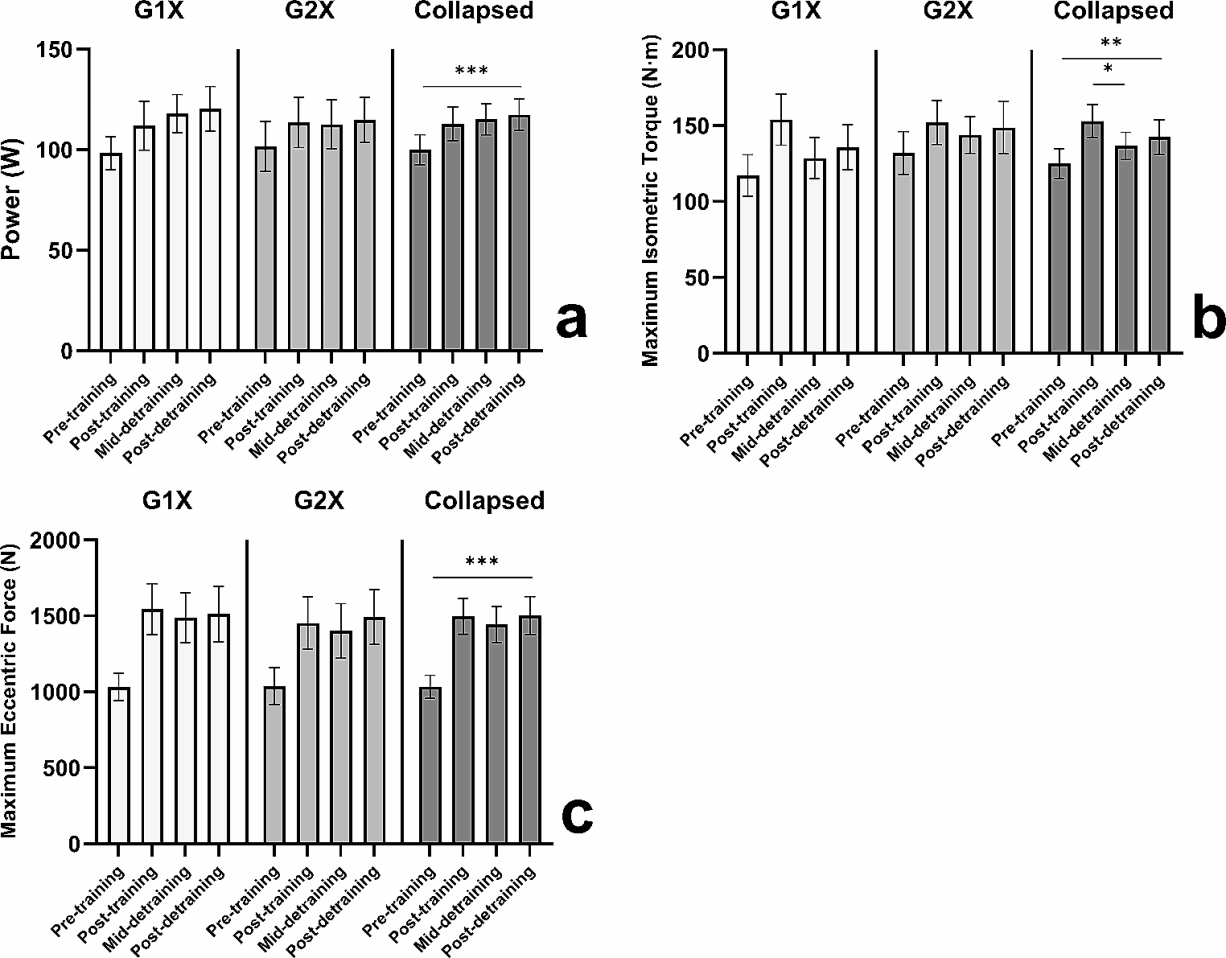
Mean ± SE for power (a), maximum isometric torque (b), and maximum eccentric force (c) at pre-training, post-training, mid-detraining, and post-detraining in the once- (G1X) and twice-weekly (G2X) training groups, alongside data collapsed by training group; where * denotes a significant difference *P* < 0.05, ** denotes a significant difference *P* < 0.01, and *** denotes a significant difference *P* < 0.001

No significant interaction effect (*F*_2, 38_ = 1.246, *P* = 0.299, η_p_^2^ = 0.082), or main effects of time (*F*_2, 38_ = 1.705, *P* = 0.195, η_p_^2^ = 0.082) nor group (*F*_1, 19_ = 0.038, *P* = 0.848, η_p_^2^ = 0.002) were revealed for power. Twelve weeks of detraining lead to 0% of the training-induced increases of power being lost, indicative of the training-induced improvements being fully retained and resulting in significantly greater power at post-detraining than at pre-training (collapsed group data = 19.5 ± 18.2% [17.4 ± 17.1 W]; *P* < 0.001, *d* = 1.02 [95% CI = 0.96, 1.09]; G1X = 22.6 ± 17.9% [22.1 ± 16.5 W], *P* = 0.002, *d* = 1.34 [95% CI = 1.25, 1.43]; G2X = 16.7 ± 18.9% [13.2 ± 17.2 W], *P* = 0.030, *d* = 0.76 [95% CI = 0.72, 0.81]), see Fig. 3a.

#### Maximum knee extensor isometric torque

No significant interaction effect was revealed for maximum isometric torque (*F*_2, 38_ = 1.623, *P* = 0.211, η_p_^2^ = 0.070), with a main effect of time (*F*_2, 38_ = 5.261, *P* = 0.010, η_p_^2^ = 0.217) but not group (*F*_1, 19_ = 0.261, *P* = 0.615, η_p_^2^ = 0.014) detected. Data collapsed across groups revealed maximum isometric torque significantly decreased from post-training to mid-detraining (-8.6 ± 15.7% [-16.4 ± 28.8 N·m]; *P* = 0.039, *d* = 0.57 [95% CI = 0.53, 0.61]), but not from mid-detraining to post-detraining (-3.5 ± 12.5% [-5.9 ± 18.6 N·m]; *P* = 0.513, *d* = 0.32 [95% CI = 0.30, 0.34]) nor from post-training to post-detraining (-6.3 ± 15.1% [-10.6 ± 27.4 N·m]; *P* = 0.241, *d* = 0.39 [95% CI = 0.36, 0.41]), indicative of the training-induced improvements being largely retained. Twelve weeks of detraining lead to 39% of the training-induced increases of isometric torque being lost, however isometric torque remained significantly greater than at pre-training (collapsed group data = (16.3 ± 20.5% [17.6 ± 23.8 N·m]; *P* = 0.001, *d* = 0.74 [95% CI = 0.69, 0.78]; G1X = 20.7 ± 25.5% [18.5 ± 29.5 N·m]; *P* = 0.044, *d* = 0.63 [95% CI = 0.59, 0.67]; G2X = 12.4 ± 14.8% [16.7 ± 18.7 N·m]; *P* = 0.024, *d* = 0.89 [95% CI = 0.84, 0.95]), see Fig. 3b.

#### Maximum lower-limb eccentric force

No significant interaction effect (*F*_2, 38_ = 0.573, *P* = 0.569, η_p_^2^ = 0.029), or main effects of time (*F*_2, 38_ = 1.452, *P* = 0.247, η_p_^2^ = 0.071) nor group (*F*_1, 19_ = 0.075, *P* = 0.787, η_p_^2^ = 0.004) were revealed for maximum eccentric force, indicative of the training-induced improvements being retained. In fact, 12 weeks of detraining lead to 0% of the training-induced increases of eccentric force being lost, resulting in significantly greater maximum eccentric force than at pre-training (collapsed group data = 46.4 ± 35.5% [468 ± 359 N]; *P* < 0.001, *d* = 1.30 [95% CI = 1.22, 1.39]; G1X = 48.4 ± 44.0% [482 ± 457 N·m]; *P* = 0.009, *d* = 1.05 [95% CI = 0.99, 1.12]; G2X = 44.5 ± 27.8% [456 ± 263 N·m]; *P* < 0.001, *d* = 1.73 [95% CI = 1.62, 1.84]), see Fig. 3c.

#### Lower-limb rate of torque development

No significant interaction effects (*F*_2, 38_ = 0.130–2.415, *P* > 0.05, η_p_^2^ = 0.007–0.113), or main effects of time (*F*_2, 38_ = 0.134–0.733, *P* > 0.05, η_p_^2^ = 0.007–0.037) nor group (*F*_1, 19_ = 0.001–0.266, *P* > 0.05, η_p_^2^ = 0.000-0.014) were revealed for RTD over all epochs or RTD_Peak_, indicative of the training-induced improvements being retained, see Table 1. However, at post-detraining when collapsed by group, whilst RTD epochs ≥ 200 ms and RTD_Peak_ were significantly greater than at pre-training (*P* < 0.05), RTD epochs ≤ 150 ms were not significantly different to pre-training (*P* > 0.05); for individual and group data of explosive metrics, see publicly available data set (10.24339/df144f2b-b9be-4b65-9c6f-65d4934b5df4).

**Table 1:** Mean ± SE rate of torque development (RTD) and contractile impulse at pre-training, post-training, mid-detraining, and post-detraining for collapsed group data

Outcome measure	Pre-training	Post-training	Mid-detraining	Post-detraining
RTD_0 − 100_ (N‧m‧s^− 1^)	451 ± 48	457 ± 51	471 ± 57	483 ± 54
RTD_0 − 150_ (N‧m‧s^− 1^)	427 ± 39	465 ± 39	489 ± 43	494 ± 45
RTD_0 − 200_ (N‧m‧s^− 1^)	368 ± 37	446 ± 40	425 ± 35	425 ± 37***
RTD_0 − 250_ (N‧m‧s^− 1^)	306 ± 27	369 ± 29	388 ± 33	386 ± 35*
RTD_0 − 300_ (N‧m‧s^− 1^)	285 ± 30	372 ± 33	354 ± 29	350 ± 32***
RTD_Peak_ (N‧m‧s^− 1^)	720 ± 92	769 ± 74	787 ± 73	785 ± 73***
Impulse_0 − 100_ (N‧m‧s)	2.42 ± 0.35	2.38 ± 0.32	2.40 ± 0.29	2.44 ± 0.27
Impulse_0 − 150_ (N‧m‧s)	5.02 ± 0.54	5.60 ± 0.54	5.56 ± 0.49	5.58 ± 0.50**
Impulse_0 − 200_ (N‧m‧s)	7.51 ± 0.74	8.98 ± 0.82	8.57 ± 0.71	8.52 ± 0.73**
Impulse_0 − 250_ (N‧m‧s)	10.26 ± 1.05	12.95 ± 1.21	12.21 ± 1.04	12.10 ± 1.09***
Impulse_0 − 300_ (N‧m‧s)	13.04 ± 1.33	16.81 ± 1.52	16.01 ± 1.30	15.80 ± 1.44***

* denotes a significant difference to pre−training *P* < 0.05, ** denotes a significant difference to pre−training *P* < 0.01, *** denotes a significant difference to pre−training *P* < 0.001

#### Lower-limb contractile impulse

No significant interaction effects (*F*_2, 38_ = 0.040–2.920, *P* > 0.05, η_p_^2^ = 0.002–0.133), or main effects of time (*F*_2, 38_ = 0.140–0.728, *P* < 0.05, η_p_^2^ = 0.007–0.037) nor group (*F*_2, 38_ = 0.012–0.227, *P* = 0.437, η_p_^2^ = 0.001–0.012) were revealed for impulse over all epochs, indicative of the training-induced improvements being retained, see Table 1. However, at post-detraining when collapsed by group, whilst contractile impulse during epochs ≥ 150 ms were significantly greater than at pre-training (*P* < 0.05), Impulse_0-100_ ms was not significantly different to pre-training (*P* > 0.05).

## Discussion

The present study examined the effects of a 12-week detraining period on the improvements in neuromuscular function and muscle structure following the cessation of once- or twice-weekly 12-week eccentric resistance training programmes. The main finding was that no significant regression occurred after 12 weeks of detraining in several neuromuscular characteristics (i.e. power, strength, and late-phase explosive capacity), resulting in values that remained significantly greater than at pre-training, which is partly in agreement with our first hypothesis. Conversely, 12 weeks of detraining was sufficient to elicit significant reductions in muscle structure, resulting in values that were not statistically greater different to pre-training. Furthermore, in agreement with our second hypothesis, no significant interaction effects were detected for any metric, indicative of similar regression profiles between once- and twice-weekly training groups.

### Neuromuscular function

Power has been suggested to be the most influential factor affecting functional ability [36] and is associated with balance [37], walking velocity [38], frailty [39], and falls [4] in older adults. Furthermore, power is more closely associated than strength with the ability to rise from a chair, stair negotiation [40], and aerobic capacity [41]. As power declines at a greater rate than muscle strength and size [3, 42], and given its association with functional ability and falls, preserving power capabilities in older adults is of major concern. The present study demonstrated that after 12 weeks of detraining, power (assessed via the STS test) did not significantly regress, with both groups retaining 100% of training-induced improvements, which were above minimum clinical threshold (9–10%) [43], resulting in values greater than at pre-training. These findings are consistent with several studies [44–47] that used traditional resistance (cyclical eccentric and concentric contractions) training and reported STS performance remained faster compared to pre-training following periods of detraining ranging from 8 to 52 weeks. Given the association between power and the ability to perform everyday tasks, it is likely that functional ability was preserved throughout the detraining period. As functional ability is a key focus of the World Health Organization’s “healthy aging” work [48], collectively these findings further reinforce the potential value of prescribing eccentric resistance training to older adults as a combative strategy to delay (or reverse) the age-related decline in functional capacity.

In the present study, no significant regression in isometric or eccentric muscular strength occurred from post-training to post-detraining, resulting in values that were significantly greater than at pre-training (isometric strength = 114% of pre-training values [61% retainment of training-induced improvements]; eccentric strength = 145% of pre-training values [100% retainment]) in both training groups, which may explain the preservation of power. The greater preservation of eccentric strength, compared to isometric, is unsurprising given it is preserved to a greater extent in older adults [49, 50], likely due to passive elements contributing towards force production. The sustainment of strength may be due to neural contributions as training-induced structural adaptations regressed within the present study (discussed below) and previous research has demonstrated that neural adaptations are sustained to a greater extent when compared to muscular and tendonous structural adaptations [51, 52]. It should, however, be noted that the superior preservation of eccentric strength may be a result of training-specific adaptations given that eccentric training was performed. The sustainment of isometric strength is also of importance regarding fall risk as weaker isometric knee extensor strength is associated with fallers (fall risk for those with greater knee extensor strength odds ratio = 0.74, *P* = 0.016) when compared to non-fallers [53], and a considerable amount (13%) of falls occur during quiet standing amongst older adults in residential settings [54]. Similarly, the preservation of lower-limb eccentric strength during aging is also important as it is required to control the body in line with gravity. For older adults, stair negotiation can be a challenging task both psychologically (i.e. fear of falling) and physically as it is performed unilaterally and requires greater lower-limb strength than level walking [55] and is associated with falls among older adults [56, 57], especially during descent [58]. Collectively, these findings are indicative that eccentric resistance training provides a potent and sustained improvement in isometric and eccentric muscular strength for at least 12 weeks following the cessation of training with important implications for exercise prescription to mitigate or prevent functional decline in older adults throughout periods of reduced physical activity.

The sustained improvements in maximum strength likely explain the preservations in late-phase explosive capacity, given the association between late-phase RTD and maximum strength [59, 60]. Explosive capacity can return to pre-training values after periods of detraining in as little as four weeks following traditional resistance training methods [17], which further emphasizes the importance of the 12-week sustainment in RTD following eccentric-only resistance training in the present study. The sustained increase in RTD (slope of the torque-time trace), resulted in an increase in contractile impulse, which is a pivotal finding given the impulse-momentum relationship and is indicative that participants may be able to swing the limb faster and more forcefully, and thus, display a greater ability to counteract a fall [61–63]. It should, however, be noted that very-early phase RTD (< 100 ms) displayed large variability and was not included in the analysis, which may be more influential on the ability to recover from a slip, trip, or fall [64], which we acknowledge is a limitation of the present study. Collectively, as maximum strength and explosive capacity are associated with the risk of falling, these data are indicative that the sustained improvements in known falls risk factors following the cessation of eccentric training likely reduced fall risk compared to pre-training, even after 12 weeks of detraining.

### Muscle structure

Unlike the sustained improvements in neuromuscular function, significant regression in muscle thickness (10%, *d =* 1.32) and fascicle angle (~ 2°, *d* = 0.73) occurred after 12 weeks of detraining, resulting in values that were not significantly different to pre-training, which likely contributed towards the slight (non-significant) decreases in isometric strength. Kay et al [25]. also reported a significant decrease in muscle size throughout detraining in the absence of a significant decrease in muscle strength, however unlike the present study, muscle size remained significantly greater than at pre-training; again, these findings are indicative of neurological adaptations contributing towards the preservation of training-induced strength adaptations, which warrant further investigation, particularly after eccentric resistance training. While the regression of muscle size and structure did not result in significant reductions in neuromuscular function, the importance of preserving muscle mass in older adults should not be overlooked. Low muscle size/quantity alongside low strength confirms a sarcopenia diagnosis [65] and given that sarcopenia is a growing public health concern due to its association with physical disability, depression, hospitalization, osteoporosis [66], and increased medical costs [6], further investigation is required to explore whether training-induced adaptations in muscle size can be sustained following the cessation of training. One potential method to preserve muscle size following the completion of a training programme may be to implement a maintenance phase, characterized by a lower dose than the initial training programme. Whilst the once- and twice-weekly training [27] and detraining (present study) demonstrated comparable profiles, indicative of a potentially minimal dosage training programme, issues with adherence may need to be considered if the maintenance dosage is too infrequent (e.g. bi-weekly or monthly). Consequently, future work is required to examine and develop a strategy that preserves muscle size and patient adherence following cessation of the initial training programme.

### Limitations

A limitation of the present study was the potential influence of changes to habitual physical activity that could have influenced the regression profiles. Despite an attempt to monitor physical activity via a commercially available triaxial accelerometer, poor compliance with wearing a physical activity monitor throughout the 26-week period (testing, training, and detraining) resulted in the inability to confidently provide meaningful data and thus, statistical analyses were not conducted. Participants were advised to wear the accelerometer on the hip to improve step count accuracy [67, 68], however given the duration of the training and detraining periods, the lack of regular contact with the researcher during the detraining period, and possibly the placement of the device (not as visible as wrist-worn), poor compliance (~ 63% of participants registered weekly step count data throughout detraining) was noted and the resultant incomplete data set was not used for analysis. The regression (or lack of) of adaptations may have been influenced by changing daily activity levels, which could have increased because of improved functional capacity or altered attitudes towards physical activity following the training intervention [69]. Although participants were instructed to maintain normal living conditions it is acknowledged that due to the duration of the detraining period (12 weeks), participants may not have fully complied with these instructions and further research is needed to develop a more effective strategy to monitor physical activity in older adults during pre-, post-, and detraining periods. A second potential limitation was that the participants in this study were community-dwelling older adults who were functionally capable, whereas clinical populations with physical and/or cognitive impairments may display different temporal profiles during training, and consequently during detraining periods due to differences in habitual activity levels [70]. Furthermore, we acknowledge the limited sample size within the present study and advise that future detraining studies recruit participant numbers with the consideration of attrition during detraining as well as training. Future research is needed to determine the most effective method to collect long-term objective physical activity data in older cohorts and to examine detraining regression profiles in those with comorbidities.

## Conclusion

Eccentric resistance training elicits substantial and durable improvements in power and maximum and explosive strength, which are sustained for at least 12 weeks after training cessation in older adults. To the authors’ knowledge, this is the first study to examine a detraining period longer than eight weeks following eccentric-only resistance training in older adults that concurrently compared the influence of initial weekly training frequency. Weekly training frequency appeared to have minimal impact upon the regression of training-induced adaptations even when one weekly training session (total weekly training time of 12 min) was performed in functionally capable community-dwelling older adults. However, given the moderate interaction effect size, further research is warranted with a larger sample size to confirm this conclusion. These findings further emphasize the potential for eccentric training to be considered an efficacious and efficient therapeutic strategy to combat neuromuscular decline in older adults. Importantly, muscle size and fascicle angle were not preserved throughout detraining and thus, further research is warranted to develop a strategy to minimize regression in muscle structure, which may be achievable by employing a maintenance dose training strategy. Given the physical, emotional, and economic cost of muscle weakness and functional impairment, further research is warranted to (i) investigate the training and detraining effects of eccentric resistance exercise on clinical populations, e.g. those diagnosed with sarcopenia or frailty, (ii) further understand the chronic temporal profile of neuromuscular adaptations following a period of eccentric resistance training longer than 12 weeks in older adults, and (iii) examine strategies that may minimize regression in muscle size and structure following the initial training period.

## Data Availability

The dataset that supports the findings of this study are openly available on PURE 10.24339/df144f2b-b9be-4b65-9c6f-65d4934b5df4.
